# ElyC and Cyclic Enterobacterial Common Antigen Regulate Synthesis of Phosphoglyceride-Linked Enterobacterial Common Antigen

**DOI:** 10.1128/mBio.02846-21

**Published:** 2021-11-23

**Authors:** Ashutosh K. Rai, Joseph F. Carr, David E. Bautista, Wei Wang, Angela M. Mitchell

**Affiliations:** a Department of Biology, Texas A&M University, College Station, Texas, USA; b Institute for Integrative Genomics, Princeton University, Princeton, New Jersey, USA; University of Georgia

**Keywords:** biosynthesis, enterobacterial common antigen, isoprenoids, metabolic regulation, outer membrane

## Abstract

The Gram-negative cell envelope is a complex structure delineating the cell from its environment. Recently, we found that enterobacterial common antigen (ECA) plays a role maintaining the outer membrane (OM) permeability barrier, which excludes toxic molecules including many antibiotics. ECA is a conserved carbohydrate found throughout *Enterobacterales* (e.g., Salmonella, Klebsiella, and *Yersinia*). There are two OM forms of ECA (phosphoglyceride-linked ECA_PG_ and lipopolysaccharide-linked ECA_LPS_) and one periplasmic form of ECA (cyclic ECA_CYC_). ECA_PG_, found in the outer leaflet of the OM, consists of a linear ECA oligomer attached to phosphoglyceride through a phosphodiester linkage. The process through which ECA_PG_ is produced from polymerized ECA is unknown. Therefore, we set out to identify genes interacting genetically with ECA_PG_ biosynthesis in Escherichia coli K-12 using the competition between ECA and peptidoglycan biosynthesis. Through transposon-directed insertion sequencing, we identified an interaction between *elyC* and ECA_PG_ biosynthesis. ElyC is an inner membrane protein previously shown to alter peptidoglycan biosynthesis rates. We found Δ*elyC* was lethal specifically in strains producing ECA_PG_ without other ECA forms, suggesting ECA_PG_ biosynthesis impairment or dysregulation. Further characterization suggested ElyC inhibits ECA_PG_ synthesis in a posttranscriptional manner. Moreover, the full impact of ElyC on ECA levels requires the presence of ECA_CYC_. Our data demonstrate ECA_CYC_ can regulate ECA_PG_ synthesis in strains wild type for *elyC*. Overall, our data demonstrate ElyC and ECA_CYC_ act in a novel pathway that regulates the production of ECA_PG_, supporting a model in which ElyC provides feedback regulation of ECA_PG_ production based on the periplasmic levels of ECA_CYC_.

## INTRODUCTION

The Gram-negative envelope is a complex multilayered structure comprised of the outer membrane (OM), the inner membrane (IM), and the periplasm containing a thin peptidoglycan layer (1, 2). The lipid component of the OM consists of an outer leaflet containing mainly lipopolysaccharide (LPS) and an inner leaflet containing phospholipids. A highly compact hydrophobic layer and highly hydrophilic layer formed by LPS, as well as the presence of transenvelope efflux pumps, render the OM impermeable to both hydrophobic molecules and large hydrophilic molecules (1, 3, 4).

The surge of antibiotic resistance in Gram-negative bacteria, especially in *Enterobacterales* (e.g., Escherichia coli, Klebsiella pneumoniae, and Salmonella sp.) has led to classification of five groups of *Enterobacterales* as urgent or serious threats by the Centers for Disease Control and Prevention (USA) (5–8). However, the study of the Gram-negative envelope, and specifically OM biogenesis, has led to the discovery of several antimicrobials in recent years (reviewed in references 9 and 10). Several antimicrobials have been identified targeting essential pathways in OM biogenesis including LPS biogenesis, protein secretion, OM protein biogenesis, and lipoprotein biogenesis (11–20). The continued success of this approach requires greater understanding of cell envelope biogenesis.

Enterobacterial common antigen (ECA) is a carbohydrate antigen present in the outer leaflet of the OM and in the periplasm and is conserved throughout *Enterobacterales* (reviewed in reference 21). The function of this molecule has remained largely unknown, in part because the biosynthesis pathways for ECA, O-antigen, and peptidoglycan overlap and in part because there are three forms of ECA that cannot currently be genetically separated (see Fig. S1 in the supplemental material). In many *Enterobacterales*, deleting the first gene in ECA biosynthesis, *wecA*, not only prevents ECA biosynthesis but also prevents O-antigen biosynthesis and increases precursor availability for peptidoglycan biosynthesis (22–25). Deletion of downstream genes in ECA biosynthesis, such as *wecE* or *wecF*, leads to accumulation of intermediates in ECA biosynthesis, interfering with peptidoglycan biosynthesis, altering cell shape, increasing envelope permeability, and activating envelope stress response systems (26–30). Three forms of ECA, LPS-linked ECA (ECA_LPS_), cyclic ECA (ECA_CYC_), and phosphoglyceride-linked ECA (ECA_PG_), are made from polymerized ECA chains. As many of the genes responsible for the steps in ECA biosynthesis separating these molecules are unknown (see below), assigning functions to these separate forms remains difficult. Nevertheless, it has become clear that in Salmonella sp., ECA plays a role in acid and bile salt resistance (31, 32) and is necessary for pathogenesis in a mouse model (32–35). In addition, we have discovered a role for ECA_CYC_ in maintaining the OM permeability barrier in E. coli (36).

10.1128/mBio.02846-21.1 FIG S1 A schematic representation of the ECA and peptidoglycan biogenesis pathways. These pathways compete for isoprenoid carrier (Und-P) and UDP-GlcNAc as the substrates. To form ECA, successive sugars are added to Und-P, and the final repeat unit is flipped across the membrane, polymerized, and made into ECA_LPS_, ECA_PG_, or ECA_CYC_. To form peptidoglycan, amino acids are attached to *N*-acetylmuramic acid, this molecule is attached to Und-P, and the final sugar is added. Then, the completed repeat unit is flipped across the IM and inserted into the peptidoglycan layer. Both pathways release Und-PP, which is recycled to yield Und-P for further synthesis. Pathway details are available in the text. G, *N*-acetylglucosamine; Ma, *N*-acetyl-d-mannosaminuronic acid; Gt, 4-acetamido-4,6-dideoxy-d-galactose; M, *N*-acetylmuramic acid. Download FIG S1, JPG file, 1.9 MB. Copyright © 2021 Rai et al. 2021 Rai et al. https://creativecommons.org/licenses/by/4.0/ This content is distributed under the terms of the Creative Commons Attribution 4.0 International license.

The polysaccharide chains of ECA consist of linear repeat units, each unit made of three sugars: GlcNAc (*N*-acetylglucosamine), ManNAcA (*N*-acetyl-d-mannosaminuronic acid), and Fuc4NAc (4-acetamido-4,6-dideoxy-d-galactose) (37, 38). Biosynthesis of ECA is initiated by attachment of GlcNAc-1-phosphate to the isoprenoid carrier, undecaprenyl-phosphate (Und-P), followed by the addition of the two remaining sugars (Fig. S1) (39–42). Und-P is a universal lipid carrier and is required for the biosynthesis of O-antigen, peptidoglycan, and capsular polysaccharides, as well as ECA (43–47). WzxE flips the complete ECA repeat unit linked to Und-P across the IM to the periplasmic face (48), and WzyE polymerizes ECA chains (49). The number of repeat units in the polymerized ECA molecule (chain length) is controlled by WzzE (50). The operon responsible for synthesis of ECA, the *wec* operon, contains the genes responsible for the steps in ECA biogenesis resulting in a polymerized ECA molecule attached to Und-PP located on the outer leaflet of the IM (39, 41, 49).

The steps through which the three forms of ECA are made from this precursor are less well understood (21). ECA_LPS_ is produced when WaaL, the O-antigen ligase, attaches ECA to the core polysaccharide of LPS (43, 51). ECA_LPS_ is presumably transported to the cell surface by the Lpt system responsible for transporting LPS to the cell surface (52). The second form, ECA_CYC_, a cyclic carbohydrate, remains in the periplasm (29). It is generally made with precise chain length (4 repeat units in E. coli K-12), and WzzE is necessary for its synthesis (29, 53, 54). The final form, ECA_PG_, is a linear ECA chain linked to diacylglycerol through a phosphodiester bond (55). The mechanism through which ECA_PG_ is formed and transported to the cell surface is completely unknown (21, 56, 57). This lack of knowledge impairs genetic studies of ECA function as mutants cannot be made that synthesize ECA_LPS_ and ECA_CYC_ in the absence of ECA_PG_.

Therefore, we set out to identify factors genetically interacting with the biosynthesis of ECA_PG_. We took advantage of the competition for substrates between the peptidoglycan and ECA biosynthesis pathways to find factors interacting with ECA_PG_ biosynthesis (Fig. S1). Using transposon-directed insertion sequencing (TraDIS), we identified ElyC as a factor interacting with ECA_PG_ biosynthesis. ElyC is an IM protein with two transmembrane domains and a large C-terminal globular DUF218 domain that resides in the periplasm (58, 59). Paradis-Bleau et al. found a Δ*elyC* mutant displays severe growth defects at low temperatures (22°C) and high frequency of cell lysis due to decreased peptidoglycan synthesis (28). They suggested that ElyC regulates the allocation of Und-P between synthesis pathways in E. coli.

Here, we have explored the role of ElyC in ECA_PG_ biosynthesis. Our data demonstrate that ElyC posttranscriptionally regulates the synthesis of ECA_PG_, greatly inhibiting its synthesis during normal growth. Furthermore, we observed that ElyC only had its full effect on ECA_PG_ synthesis in the presence of WzzE, suggesting that WzzE or ECA_CYC_ is involved in this regulatory pathway. In fact, we found that ElyC and ECA_CYC_ act together to regulate ECA_PG_ biosynthesis. Our data demonstrate that the effect of ElyC and ECA_CYC_ on ECA levels is specific to ECA_PG_ and not a result of allocation of Und-P between biosynthesis pathways. Overall, we have deciphered a novel pathway through which ElyC and ECA_CYC_ regulate ECA_PG_ biosynthesis, providing insight into the elusive function of ElyC and demonstrating that ECA_CYC_ plays roles both in maintaining the OM permeability barrier and in regulating ECA biosynthesis.

## RESULTS

### Identification of candidate genes interacting with ECA_PG_ biosynthesis.

There are genes known to be necessary for the biosynthesis of ECA_CYC_ and of ECA_LPS_ specifically: ECA_CYC_ synthesis requires *wzzE*, while ECA_LPS_ synthesis requires *waaL* (29, 36, 43). However, the genes and reactions responsible for producing ECA_PG_, by transferring the ECA polymer from Und-PP to form phosphoglyceride-linked ECA_PG_, and for its surface exposure are unknown (55–57). Therefore, we set out to identify factors involved in ECA_PG_ biosynthesis, utilizing interactions between ECA and peptidoglycan biosynthesis.

The ECA and peptidoglycan pathways compete for Und-P and UDP-GlcNAc as depicted in Fig. S1 in the supplemental material (25, 60, 61). Although deletion of *wecA* causes generally mild phenotypes, deletion of later genes in ECA biosynthesis (e.g., *wecB*, *wecG*, *wecF*, or *wzxE*) causes the accumulation of ECA intermediates sequestering Und-P, disrupting peptidoglycan biosynthesis and resulting in increased permeability defects, cell shape defects, and envelope stress response activation (26–30, 62, 63). In fact, deletion of *wzyE*, the ECA polymerase, or all the flippases capable of flipping lipid III^ECA^ across the IM is lethal (29). Thus, we hypothesized that, in a strain making only ECA_PG_, disruption or dysregulation of the next step in ECA biosynthesis (transfer of polymerized ECA from Und-PP) would also be highly unfavorable due to sequestration of Und-P inhibiting peptidoglycan biosynthesis.

**FIG 1:**
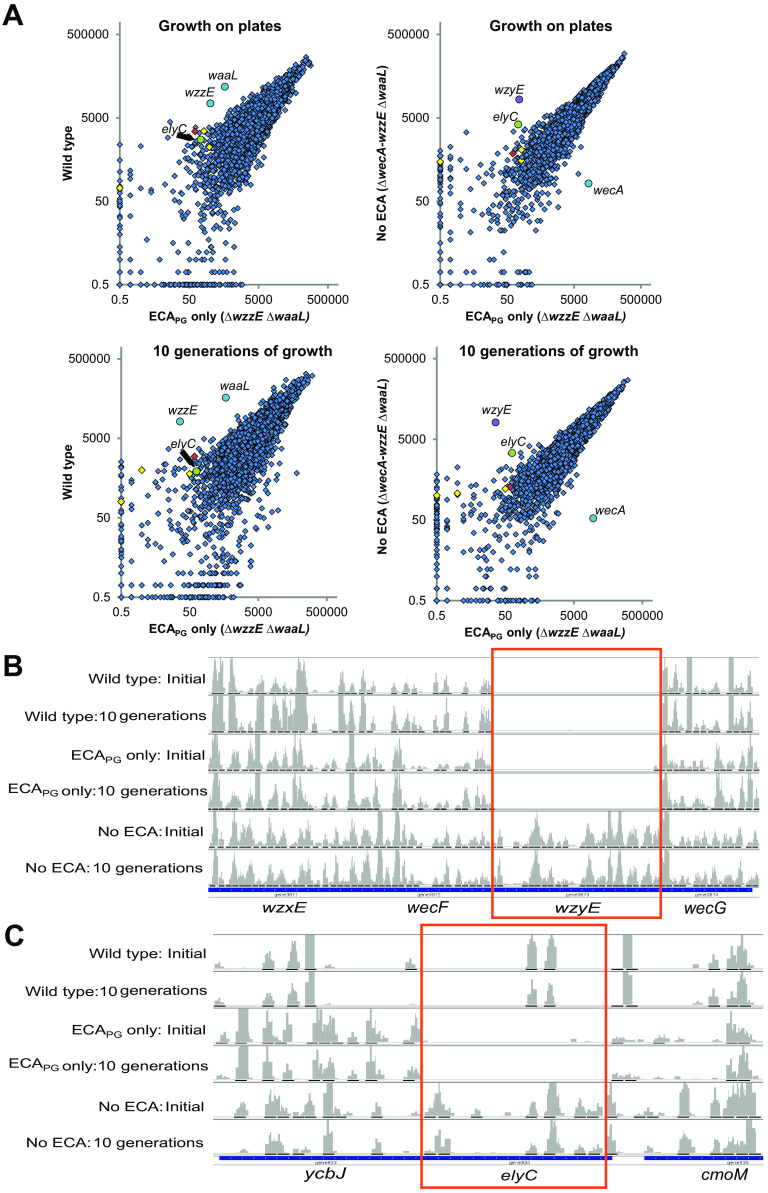
Screening for candidate genes interacting with ECA_PG_ biosynthesis. (A) TraDIS was used to identify genes for which disruption was unfavorable in cells making ECA_PG_ without the other forms of ECA. Scatterplots of transposon junction reads per gene are shown comparing the ECA_PG_ strain (Δ*wzzE* Δ*waaL*) with an isogenic strain without ECA (Δ*wecA* Δ*wzzE* Δ*waaL*) and with wild-type MG1655. Results are shown following initial growth on plates and after 10 additional generations of growth in liquid medium. Putative ECA_PG_ biosynthesis genes are shown in yellow, *elyC* is shown in green, *wzyE* is shown in purple, and *ynbB* is shown in red. Genes deleted in one of the strains are shown in cyan. (B) Histograms of transposon insertion reads in *wzyE* and adjacent genes are shown as a control for detection of changes in essentiality based on Und-P availability. Transposon insertions are observed in *wzyE* only in the strain without ECA. (C) Histograms of transposon insertion reads in *elyC* and adjacent genes. Transposon insertions are observed in the strain without ECA and the wild-type strain but not in the strain making only ECA_PG_, suggesting essentiality of *elyC* in the ECA_PG_-only strain.

Therefore, we have used TraDIS (transposon-directed insertion sequencing) (64) to compare the favorability of gene disruptions in a mutant which makes ECA_PG_ but not the other forms of ECA (Δ*wzzE* Δ*waaL*) with an isogenic mutant that does not make ECA (Δ*wecA-wzzE* Δ*waaL*) and with wild-type E. coli K-12 MG1655. For this approach, we generated high-density transposon libraries in each of these strains and performed Illumina sequencing of the transposon junctions in the initial pooled libraries, as well as after 10 generations of growth in liquid culture. The statistical properties of each data set were similar (Table S1). We then compared the transposon junction reads per gene between the three strains (Fig. 1A). To confirm we could detect changes in essentiality due to sequestration of Und-P, we analyzed the transposon junction reads in *wzyE*. In strains producing ECA, *wzyE* is essential; however, *wzyE* becomes nonessential when ECA synthesis is disrupted at an earlier step as accumulation of lipid III^ECA^ is prevented (29). In the ECA_PG_-only strain and wild-type MG1655, we detected very few transposon insertion reads in *wzyE*; however, we observed similar levels of insertions to nearby genes in the strain without ECA (Fig. 1B).

10.1128/mBio.02846-21.7 TABLE S1 Descriptive statistics for TraDIS. Download Table S1, PDF file, 0.08 MB. Copyright © 2021 Rai et al. 2021 Rai et al. https://creativecommons.org/licenses/by/4.0/ This content is distributed under the terms of the Creative Commons Attribution 4.0 International license.

To identify genes possibly involved in ECA_PG_ biosynthesis, we defined a set of criteria for genes putatively essential only when ECA_PG_ is made without the other forms of ECA (Table S2). These genes had less than 200 reads in the ECA_PG_-only library under both growth conditions and had at least a 1-standard-deviation decrease in the ECA_PG_-only strain compared to the other two strains under both growth conditions. In addition, we limited our analysis to genes not known to be essential in wild-type E. coli K-12 that make proteins targeted to either the IM or the periplasm (65–67). We identified five genes that fit these criteria: *elyC*, *ynbB*, *ymiB*, *lapA*, and *yoaI* (Table S2). From these hits, we confirmed the data in the literature that *ynbB* was not necessary for the synthesis of ECA_PG_ (68; unpublished data).

10.1128/mBio.02846-21.8 TABLE S2 Possible genes involved in ECA_PG_ biogenesis. Download Table S2, PDF file, 0.1 MB. Copyright © 2021 Rai et al. 2021 Rai et al. https://creativecommons.org/licenses/by/4.0/ This content is distributed under the terms of the Creative Commons Attribution 4.0 International license.

In this paper, we focus on *elyC* (Fig. 1C), which encodes an inner membrane protein. Previous work has shown that a Δ*elyC* mutant lyses at room temperature (22°C) due to a peptidoglycan synthesis defect but grows well at 37°C (28). The authors hypothesized this defect is due to competition between peptidoglycan and the ECA biosynthesis pathway, particularly at the step of allocation of Und-P. Data have also suggested that an Δ*elyC* mutant may have a periplasmic protein-folding defect (69) and may experience increased oxidative stress at low temperature (22°C) (70). The experiments described here were performed at 37°C.

### *elyC* is essential in a strain producing only ECA_PG_.

To determine whether *elyC* was essential in a strain producing only ECA_PG_, we performed genetic linkage-disruption experiments. In these experiments, a Tn*10* marker genetically linked to a deletion in the gene of interest is transduced into strains, selecting for the presence of Tn*10*. Based on the size of DNA packaged by the P1*vir* phage, the gene deletion is cotransduced with a calculable frequency (71). If there is selection against the deletion of the gene (i.e., the gene is essential), the cotransduction frequency observed will decrease. We first measured linkage between *zbj-7230*::Tn*10* and Δ*elyC*::kan. These two markers were approximately 53% linked in wild-type MG1655 and in Δ*waaL* and Δ*wzzE* single mutants (Table 1). However, in a *ΔwzzE* Δ*waaL* double mutant producing only ECA_PG_, we observed only 1% linkage, demonstrating strong linkage disruption (Table 1). The linkage is restored in a complemented strain. We observed similar linkage disruption when transducing *metE3074*::Tn*10* linked to Δ*wzzE*::kan into a Δ*waaL* Δ*elyC* mutant (Table 1). These data confirm that *elyC* is essential when ECA_PG_ is made in isolation but not in strains making two or more forms of ECA.

**TABLE 1:** *elyC* is essential in strain making only ECA_PG_

Donor	Recipient	Recipient form(s) of ECA	*N* ^*a*^	P1*vir* cotransduction frequency^*b*^
*zbj*-7230::Tn*10* Δ*elyC*::kan (AM769)	MG1655	ECA_CYC_, ECA_LPS_, ECA_PG_	300	52.7%
	Δ*wzzE* (AM365)	ECA_LPS_, ECA_PG_	300	54.3%
	Δ*waaL* (AM366)	ECA_CYC_, ECA_PG_	300	53.3%
	Δ*wzzE* Δ*waaL* (AM395)	ECA_PG_	300	1.0%
	Δ*wzzE* Δ*waaL* pBAD33*-elyC*^*c*^ (AM1159)	ECA_PG_	306	62.1%

*metE-*3074::Tn*10* Δ*wzzE*::kan (AM766)	MG1655	ECA_CYC_, ECA_LPS_, ECA_PG_	300	25.0%
	Δ*elyC* (AM743)	ECA_CYC_, ECA_LPS_, ECA_PG_	300	17.3%
	Δ*waaL* (AM366)	ECA_CYC_, ECA_PG_	300	24.3%
	Δ*waaL* Δ*elyC* (AM745)	ECA_CYC_, ECA_PG_	300	3.0%

*thd*::Tn*10* Δ*waaL*::kan (AM735)	MG1655	ECA_CYC_, ECA_LPS_, ECA_PG_	295	79.0%
	Δ*elyC* (AM743)	ECA_CYC_, ECA_LPS_, ECA_PG_	300	72.3%
	Δ*wzzE* (AM365)	ECA_LPS_, ECA_PG_	300	76.0%
	Δ*wzzE* Δ*elyC* (AM744)	ECA_LPS_, ECA_PG_	232	61.2%

a The indicated number of transductants were analyzed. Transductants were harvested from three separate transductions.

b P1*vir* was used to transduce the indicated markers into the indicated strain. Cotransduction frequency was determined by selecting the transductants for the presence of Tn*10* and calculating the percentage of colonies containing the gene deletion.

c Expression from complementing plasmid was induced with 0.2% arabinose.

Interestingly, we observed only slight linkage disruption when transducing *thd*::Tn*10* Δ*waaL*::kan into a Δ*wzzE* Δ*elyC* strain (Table 1). We confirmed these results by rebuilding the strains from wild-type MG1655 and with two different alleles of Δ*elyC* (Table S3) and through direct transduction of the Δ*waaL*::kan allele. Although the triple deletion mutant, Δ*wzzE* Δ*elyC* Δ*waaL*::kan *thd*::Tn*10*, could be built with Δ*waaL* as the last deletion, the triple mutant colony size was extremely small compared to Δ*wzzE* Δ*elyC thd*::Tn*10* colonies (Fig. S2). These data suggest that, although *elyC* is essential in a strain producing only ECA_PG_, its function is somehow modified in a Δ*wzzE* strain allowing survival when *waaL* is deleted last (see below).

10.1128/mBio.02846-21.2 FIG S2 Loss of *waaL* impedes growth in a Δ*wzzE* Δ*elyC* background. Δ*wzzE* Δ*elyC* cells were transduced with a P1*vir* lysate from a *tdh*::Tn*10* Δ*waaL*::kan strain, selecting for the presence of the Tn*10*. Representative Δ*wzzE* Δ*elyC tdh*::Tn*10* colonies are marked with black arrows. Representative Δ*wzzE* Δ*elyC* Δ*waaL*::kan *tdh*::Tn*10* colonies are marked with blue arrows. The presence of Δ*waaL*::kan was confirmed through kanamycin resistance. The triple-deletion cells showed greatly reduced growth compared to the isogenic controls. Download FIG S2, JPG file, 1.3 MB. Copyright © 2021 Rai et al. 2021 Rai et al. https://creativecommons.org/licenses/by/4.0/ This content is distributed under the terms of the Creative Commons Attribution 4.0 International license.

10.1128/mBio.02846-21.9 TABLE S3 Linkage disruption with deletion of *waaL* Table S3, PDF file, 0.1 MB. Copyright © 2021 Rai et al. 2021 Rai et al. https://creativecommons.org/licenses/by/4.0/ This content is distributed under the terms of the Creative Commons Attribution 4.0 International license.

### Deletion of *elyC* increases ECA_PG_ levels.

**FIG 2:**
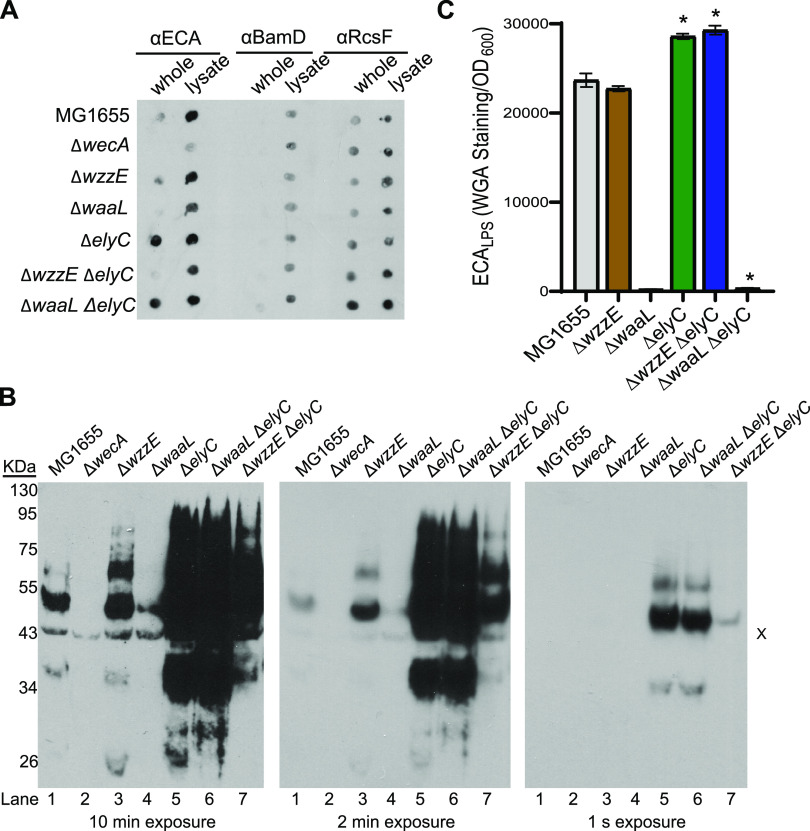
Deletion of *elyC* increases levels of ECA_PG_. (A) The surface exposure of ECA_PG_ and ECA_LPS_ was detected through dot blot assay. Whole cells or a whole-cell lysate was probed for ECA, BamD, or RcsF. BamD acted as a negative control for surface exposure, while RcsF acted as a positive control for surface exposure. Δ*wecA* served as a negative control for the presence of ECA. Surface-exposed ECA was detected in all Δ*elyC* strains including the Δ*waaL* Δ*elyC* strain, suggesting ECA_PG_ is present on the cell surface in these strains. (B) Immunoblotting was performed to examine ECA levels and chain length. A very large increase in ECA levels was observed in Δ*elyC* and Δ*waaL* Δ*elyC* strains, but less of an increase was observed in the Δ*wzzE* Δ*elyC* strain. The nonspecific “X” band serves as a loading control. Δ*wecA* serves as a negative control for the presence of ECA. (C) ECA_LPS_ quantification was performed in indicated strains by WGA staining. Data are shown as fluorescence relative to OD_600_. The Δ*waaL* and Δ*waaL* Δ*elyC* strains serve as negative controls for the presence of ECA_LPS_. There was a small but significant increase in ECA_LPS_ levels in the Δ*elyC* and Δ*wzzE* Δ*elyC* strains compared to their parent strains. Data are shown as the mean from three biological replicates ± standard error of the mean (SEM). *, *P* < 0.05 by the nonparametric Mann-Whitney test compared to *elyC*^+^ parent strain.

After confirming *elyC*’s essentiality in a strain making only ECA_PG_, we asked what the effect of the Δ*elyC* mutation was on surface exposure of ECA, as a Δ*waaL* strain without ECA_PG_ should not have ECA on its surface. We used a dot blot as a qualitative method (72) to detect surface-exposed ECA_PG_ and ECA_LPS_. ECA_CYC_ is not surface exposed. Surface-exposed ECA was detected in all Δ*elyC* strains including the Δ*waaL* Δ*elyC* strain (Fig. 2A). In fact, the surface-exposed ECA levels appeared higher in the Δ*elyC* and Δ*waaL* Δ*elyC* strains than in the wild-type or Δ*wzzE* Δ*elyC* strains. These observations suggested that there might be an increase in a non-ECA_LPS_, surface-exposed species of ECA in the Δ*elyC* mutant. As dot blots are not ideal for determining quantitative changes, we performed ECA immunoblot analyses to detect the charged forms of ECA (ECA_PG_ and ECA_LPS_). ECA_CYC_ is not charged and cannot be observed through immunoblot analysis. We found a very large increase in linear ECA levels in both the Δ*elyC* and Δ*waaL* Δ*elyC* strains (Fig. 2B; compare lanes 5 and 6 with lanes 1 and 4). Similar to the dot blot results, there was much less of an increase in ECA levels in the Δ*wzzE* Δ*elyC* strain (lane 7). These results suggest, when *elyC* is deleted, there is a large increase in a species of ECA which is neither ECA_LPS_ nor ECA_CYC_.

Thus, we sought to determine ECA_LPS_ levels. Wheat germ agglutinin (WGA) is a lectin protein used to detect glycans (β-GlcNAc or sialic acid multimers) in prokaryotes and eukaryotes (73–78). A beta-linked GlcNAc is present in the glycosidic bond that attaches ECA to LPS to form ECA_LPS_, but this bond is absent in ECA_PG_. Therefore, we have found cell surface staining of MG1655 with WGA labels only ECA_LPS_, providing a specific assay for this ECA species (Fig. S3A). Thus, we assayed WGA staining of *elyC* mutant cells and found deletion of *elyC* caused only a slight increase in the amount of ECA_LPS_. This increase was similar between the Δ*elyC* and Δ*wzzE* Δ*elyC* strains (Fig. 2C). There are two possible explanations for the smaller increase in ECA_LPS_ levels than linear ECA levels: (i) ElyC plays a role in ECA biosynthesis that is specific to ECA_PG_ or (ii) ECA_LPS_ levels are limited by availability of WaaL. Therefore, we overexpressed *waaL* in the wild-type and Δ*elyC* strains and assayed levels of ECA_LPS_. Although we observed an increase in ECA_LPS_ levels when *waaL* was overexpressed in a wild-type strain, we did not see an increase in ECA_LPS_ level in the Δ*elyC* strain (Fig. S4A), demonstrating that Δ*elyC* is epistatic to *waaL* expression. These data suggest that the effect of ElyC is specific to ECA_PG_ biosynthesis. Overall, the immunoblot, dot blot, and WGA staining experiments demonstrate that ElyC plays a role in ECA_PG_ biosynthesis that leads to a large increase in the levels of a non-ECA_LPS_ species with deletion of *elyC*.

10.1128/mBio.02846-21.3 FIG S3 WGA staining and *elyC* overexpression validation. (A) Live cells of the indicated genotypes were stained with WGA conjugated to the Alexa Fluor 488 fluorophore, and their relative fluorescence was measured. No WGA sample denotes mock-stained wild-type control cells. Staining was observed in the wild-type strain and a Δ*wzzE* strain lacking ECA_CYC_. However, no staining was observed in the Δ*wecA*, Δ*waaL*, and Δ*wzzE* Δ*waaL* strains that lack ECA_LPS_. Data are shown as the mean from three biological replicates ± SEM. (B) ECA_LPS_ quantification was performed in a Lac^−^ strain (MC4100 Ara^R/−^) overexpressing *elyC* from the IPTG-inducible pCA24N vector. Overexpressing *elyC* decreased ECA_LPS_ levels. pV sample contains the strain with empty pCA24N treated with 100 μM IPTG. Data are shown as the mean from three biological replicates ± SEM. (C) ECA_PG_ levels were assayed in the Δ*wzzE* Δ*waaL* double mutant with empty pBAD33 and pBAD33-*elyC* induced by arabinose. *elyC* overexpression resulted in decreased ECA_PG_ levels. The nonspecific “X” band serves as a loading control. (D) ECA_LPS_ levels were measured in MG1655 and the Δ*wzzE* mutant strain in the presence of empty pBAD33 and pBAD33-*elyC* induced by arabinose or repressed by α-d-fucose. ECA_LPS_ levels decreased with *elyC* overexpression in both strains. Representative data are shown as fold values relative to the vector control under the same induction conditions. Download FIG S3, JPG file, 0.06 MB. Copyright © 2021 Rai et al. 2021 Rai et al. https://creativecommons.org/licenses/by/4.0/ This content is distributed under the terms of the Creative Commons Attribution 4.0 International license.

10.1128/mBio.02846-21.4 FIG S4 Overexpression of *waaL* and *murA*. (A) *waaL* was overexpressed from the IPTG-inducible pCA24N vector in wild-type and Δ*elyC* strains, and ECA_LPS_ levels were measured using WGA staining. In wild-type cells, increasing overexpression of *waaL* causes significant increases in ECA_LPS_ levels. In Δ*elyC* cells, increasing *waaL* overexpression does not increase ECA_LPS_ levels, demonstrating that Δ*elyC* is epistatic to *waaL* overexpression for ECA_LPS_ levels. Data are shown as the mean from three biological replicates ± SEM. *, *P* < 0.05 by the Mann-Whitney test. (B) Linear ECA levels were assayed by immunoblotting in MG1655 strains harboring empty pCA24N (pV), pCA24N-*elyC*, or pCA24N-*murA* plasmids induced with different IPTG concentrations (0, 10, 25, and 100 μM IPTG). The 100 μM IPTG sample was omitted for pCA24N-*elyC* due to toxicity. Both *murA* and *elyC* overexpression decrease linear ECA levels. Download FIG S4, JPG file, 0.06 MB. Copyright © 2021 Rai et al. 2021 Rai et al. https://creativecommons.org/licenses/by/4.0/ This content is distributed under the terms of the Creative Commons Attribution 4.0 International license.

### ElyC posttranscriptionally regulates the production of ECA_PG_ and ECA overall.

There are two possible models to explain the increase in ECA observed when *elyC* is deleted. First, ElyC may decrease ECA_PG_ levels by inhibiting the production of ECA_PG_. Second, there might be more than one step necessary to produce ECA_PG_ and ElyC is responsible for a later step leading to the accumulation of a biosynthetic intermediate that is not distinguishable from ECA_PG_ on an immunoblot. To differentiate between these models, we determined the effect of overexpressing *elyC* on ECA levels. In the first model, overexpression of ElyC should decrease ECA_PG_ levels, while, in the second model, overexpression of ElyC should either not effect or increase production of ECA_PG_, depending on the rate-limiting step in synthesis.

**FIG 3:**
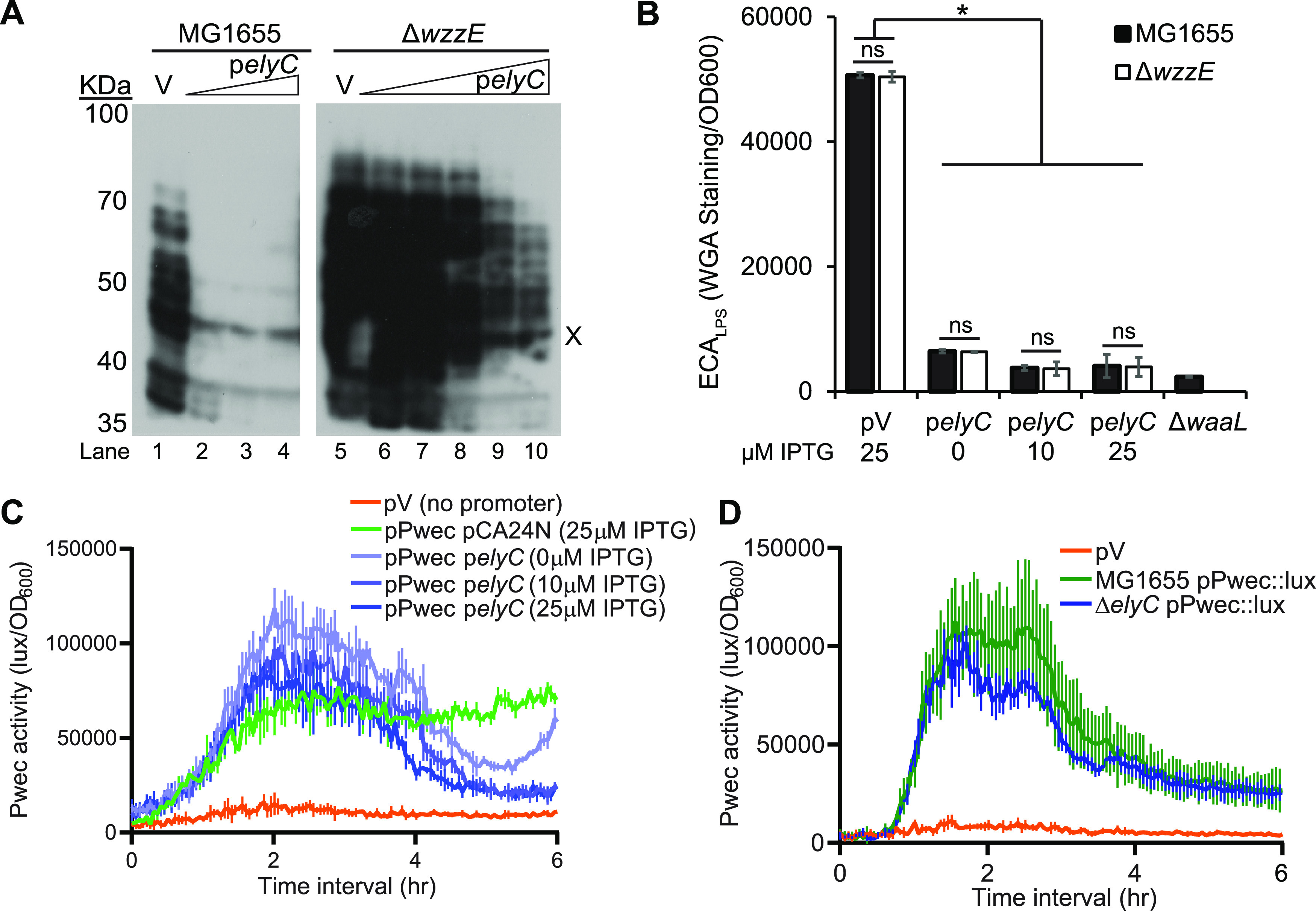
ElyC regulates ECA_PG_ production posttranscriptionally. (A to C) *elyC* was overexpressed from the IPTG-inducible pCA24N vector in the indicated strains. (A) Triangles indicate increasing overexpression of *elyC*. In wild-type cells, even low-level overexpression of *elyC* greatly decreased ECA levels; however, less of a decrease was observed in the Δ*wzzE* strain. The nonspecific “X” band serves as a loading control. “V” samples indicate strains with empty pCA24N induced with 100 μM IPTG. Lanes 2 to 4 are induced with 0, 10, and 25 μM IPTG, respectively. Lanes 6 to 10 are induced with 0, 10, 25, 50, and 100 μM IPTG, respectively. (B) ECA_LPS_ quantification was performed by WGA staining, indicated by fluorescence relative to OD_600_. The Δ*waaL* strain acts as a negative control. Overexpression of *elyC* reduced ECA_LPS_ levels, but no difference was observed between the wild-type strain and the Δ*wzzE* strain. (C) A bacterial luciferase reporter was used to assay the activity of the P*_wec_* promoter, the promoter for the *wec* operon containing genes for ECA biosynthesis. Despite the decrease in ECA levels observed, overexpression of *elyC* did not decrease P*_wec_* activity. (D) P*_wec_* activity was assayed as in panel C. No increase in P*_wec_* activity was observed in the Δ*elyC* strain compared to the wild-type strain. Quantitative data are shown as the mean from three biological replicates ± SEM. *, *P* < 0.05 by the nonparametric Mann-Whitney test; ns, *P* > 0.05 by the Mann-Whitney test.

We assayed ECA levels by immunoblotting in strains overexpressing *elyC* in a wild-type or Δ*wzzE* background. We utilized the pCA24N-*elyC* plasmid from the ASKA collection, which expresses *elyC* under a leaky, isopropyl-β-d-thiogalactopyranoside (IPTG)-inducible promoter (79). We observed that increasing *elyC* overexpression in a wild-type background greatly decreased ECA levels (Fig. 3A, lanes 1 to 4). In a Δ*wzzE* mutant, a smaller decrease in ECA levels was observed at high IPTG concentrations (lanes 5 to 10). As with our previous results, this suggests that the removal of *wzzE* changes ElyC’s effect on ECA levels. To assay the effect of *elyC* overexpression on ECA_LPS_ in the wild-type and Δ*wzzE* strains, we performed WGA staining and observed a decrease in ECA_LPS_ levels that was similar in the two backgrounds (Fig. 3B). We observed similar results when *elyC* was overexpressed in MC4100 (a Lac^−^ strain) (Fig. S3B) and when *elyC* was overexpressed from a pBAD33 plasmid (Fig. S3C and D). Overall, these results demonstrate that overexpressing *elyC* decreases ECA levels and ElyC is responsible for inhibiting the production of ECA_PG_ and, to some extent, ECA production overall.

We then asked whether *elyC* affects ECA levels on a transcriptional or posttranscriptional level. We constructed a reporter by cloning the promoter region of the *wec* operon into a promoterless pJW15 vector that harbors a bacterial luciferase operon (Fig. S5) (80, 81). Using this reporter, we observed no consistent decrease in P*_wec_* activity with *elyC* overexpression, despite the decrease in ECA levels (Fig. 3C). We also checked the effect of *elyC* deletion on P*_wec_* activity and found no increase the P*_wec_* reporter activity in this strain (Fig. 3D). Overall, we observed no indication that ElyC regulates ECA levels in a transcriptional manner, making posttranscriptional regulation most likely.

10.1128/mBio.02846-21.5 FIG S5 Vector map of pJW15-P*_wec_*. The region of P*_wec_* promoter, which drives expression of the *wec* operon responsible for many steps in ECA biosynthesis, from −500 to +20 relative to the *wecA* translational start site was cloned into the pJW15 vector between the EcoRI and BamHI restriction sites. The P*_wec_* promoter drives the expression of the *luxCDABE* genes causing luminescence when the P*_wec_* promoter is active. Adapted from data from the work of Wong et al. (J. L. Wong, S. L. Vogt, and T. L. Raivio, Methods Mol Biol 966:337–357, 2013, https://doi.org/10.1007/978-1-62703-245-2_21). Download FIG S5, JPG file, 0.1 MB. Copyright © 2021 Rai et al. 2021 Rai et al. https://creativecommons.org/licenses/by/4.0/ This content is distributed under the terms of the Creative Commons Attribution 4.0 International license.

### ECA_CYC_ acts with ElyC to regulate ECA_PG_ production.

**FIG 4:**
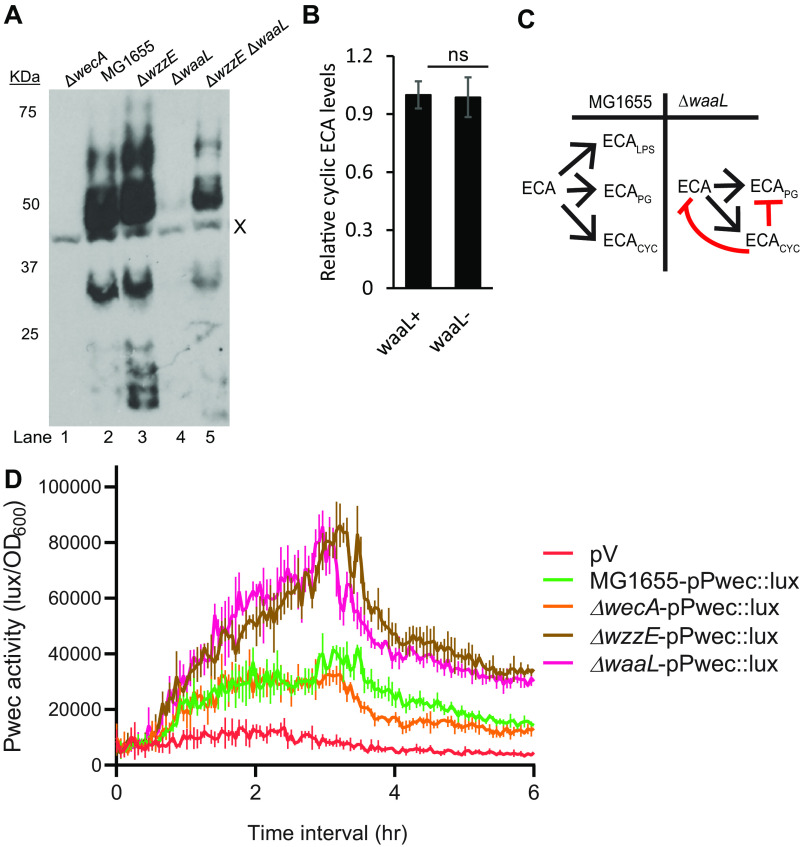
ECA_CYC_ is involved in feedback regulation of ECA_PG_ levels. (A) The level of the linear forms of ECA was assayed in the indicated strains by immunoblotting. The Δ*wecA* strain serves as a negative control for ECA. The nonspecific “X” band serves as a loading control. Much less ECA_PG_ is observed in the Δ*waaL* strain compared to linear ECA levels in the wild-type strain. However, in the Δ*wzzE* Δ*waaL* strain, ECA_PG_ levels return to near-wild-type levels. (B) ECA_CYC_ levels were compared in Δ*wecH*::kan and Δ*waaL* Δ*wecH*::kan strains by MALDI-TOF. WecH is responsible for nonstoichiometric acetylation of ECA. The levels of ECA_CYC_ are comparable between the strains. (C) Model for ECA_PG_ and ECA_CYC_ levels with loss of ECA_LPS_ is shown. In wild-type cells, the three forms of ECA are produced at an appropriate ratio. When Δ*waaL* is deleted and ECA_LPS_ is lost, the remaining ECA is funneled into ECA_PG_ and ECA_CYC_. However, ECA_CYC_ levels are maintained at a consistent level and ECA_PG_ levels are decreased, suggesting ECA_CYC_ levels are measured to provide regulation of ECA_PG_ production and ECA levels overall (red arrows). (D) Activity of the P*_wec_* promoter was assayed by luciferase assay in the indicated strains. P*_wec_* activity does not correlate with changes in ECA levels, suggesting that the effect of ECA_CYC_ on ECA levels is posttranscriptional. Quantitative data are shown as the mean from three biological replicates ± SEM. ns, *P* > 0.05 by the Mann-Whitney test.

Throughout our experiments, we observed that the effect of ElyC on ECA_PG_ levels was less in the absence of *wzzE*. This led us to ask whether WzzE or ECA_CYC_ was playing a role in the pathway through which ElyC regulated ECA_PG_ levels. To differentiate between the effects of WzzE and ECA_CYC_ on this pathway, we utilized the previous observation that levels of linear ECA are very low in a Δ*waaL* mutant (36). We have confirmed this effect: there is much less ECA detectable by immunoblotting in Δ*waaL* cells than in wild-type cells or a Δ*wzzE* mutant (Fig. 4A; lane 4 compared to lanes 2 and 3). However, in the Δ*wzzE* Δ*waaL* mutant the ECA levels return to near-wild-type levels (lane 5). Our initial explanation was that there was much more ECA_LPS_ than ECA_PG_ present and that the excess ECA freed by removing ECA_LPS_ was funneled into ECA_CYC_, which is not detectable by immunoblotting. However, this did not fit well with our observations of overexpressing *elyC* in a Δ*wzzE* background (Fig. 3A and B), where we observed a large decrease in ECA_LPS_ levels but a relatively small decrease in ECA levels overall. Therefore, we purified ECA_CYC_ and quantified the ECA_CYC_ levels through matrix-assisted laser desorption ionization–time of flight (MALDI-TOF) mass spectroscopy (Fig. S6). We found no effect of Δ*waaL* on cyclic ECA levels (Fig. 4B and Fig. S6). Thus, while the ECA_CYC_ levels remained constant, the total amount of ECA was decreased in the Δ*waaL* strain. That a free pool of ECA caused by the removal of ECA_LPS_ leads to decreased linear ECA levels and steady ECA_CYC_ levels demonstrates that ECA_CYC_ plays a role in regulating ECA_PG_ biosynthesis (Fig. 4C). Thus, ECA_CYC_ is involved in regulation of ECA_PG_ production. Then, we measured P*_wec_* activity in Δ*wecA*, Δ*wzzE*, and Δ*waaL* strains to determine whether the regulation is transcriptional or posttranscriptional. We found that any changes in reporter activity in the mutants did not correlate with the changes in the amounts of ECA observed (Fig. 4A and D). Thus, ECA_CYC_ has a role in controlling ECA_PG_ production that appears to be through posttranscriptional regulation. Importantly, the levels of ECA are similar between the Δ*elyC* and Δ*waaL* Δ*elyC* strains (Fig. 2B), and the effect of ElyC on ECA_PG_ levels is much less in the absence of *wzzE* (Fig. 2B and Fig. 3A). These data demonstrate that ElyC and ECA_CYC_ act together in this regulatory pathway.

10.1128/mBio.02846-21.6 FIG S6 MALDI-TOF for effect of Δ*waaL* on ECA_CYC_ levels. The Δ*wecH* Δ*waaL* strain was grown in minimal medium containing normal nitrogen (N^14^), while the control Δ*wecH* strain was grown in minimal medium containing heavy nitrogen (N^15^). Deletion of *wecH* prevents nonstoichiometric acylation of ECA. Equal amounts of cells were combined, and ECA_CYC_ was purified and subjected to MALDI-TOF. The respective peaks, Δ*wecH* Δ*waaL* (*m/z* 2427) and Δ*wecH* (*m/z* 2439), corresponding to ECA_CYC_ are labeled. The three colors represent three biological replicates. The areas of the respective peaks in each biological replicate are shown in the inset table. Download FIG S6, JPG file, 1.1 MB. Copyright © 2021 Rai et al. 2021 Rai et al. https://creativecommons.org/licenses/by/4.0/ This content is distributed under the terms of the Creative Commons Attribution 4.0 International license.

### Und-P allocation is not responsible for the effect of ElyC on ECA levels.

Previous reports have shown that the overexpression of the gene responsible for Und-P synthesis or the gene responsible for the first step in peptidoglycan biosynthesis relieved peptidoglycan stress in a Δ*elyC* strain (28, 82). This led to the suggestion that ElyC balances Und-P use between pathways (28). It is not possible for the effect of ElyC specifically on ECA_PG_ to be caused by Und-P allocation. However, it is possible that Und-P allocation is responsible for the effect on total ECA levels.

**FIG 5:**
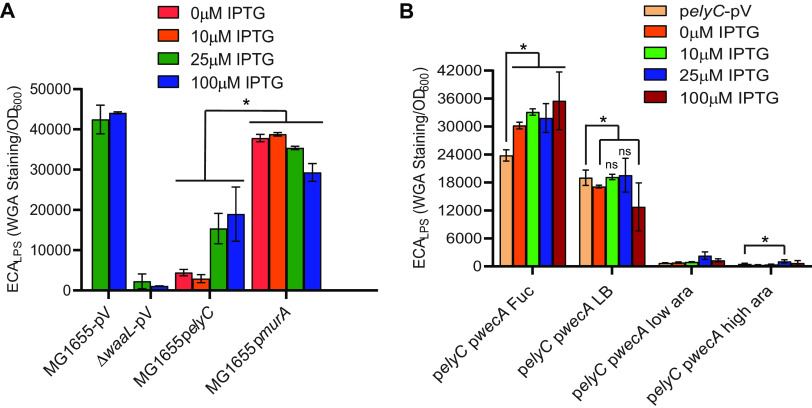
ElyC regulates ECA levels independently of Und-P availability. ECA_LPS_ levels were assayed by WGA staining. Data are shown as fluorescence relative to OD_600_. (A) *elyC* or *murA*, the first gene in peptidoglycan biosynthesis, was overexpressed from the IPTG-inducible pCA24N vector. Overexpression of *murA* increases the utilization of Und-P by the peptidoglycan biosynthesis pathway. The effect of *murA* overexpression on ECA_LPS_ levels was smaller than that of *elyC*. (B) *elyC* was overexpressed from the arabinose-inducible and fucose-repressible pBAD33(K) plasmid. *wecA* was overexpressed from the IPTG-inducible pCA24N vector. Overexpression of *wecA* increases the utilization of Und-P by the ECA biosynthesis pathway. *wecA* overexpression increases production of ECA_LPS_ in the absence of *elyC* overexpression. However, *wecA* overexpression does not suppress the decrease in ECA_LPS_ levels when *elyC* is overexpressed. Fuc, 0.05% fucose; LB, no inducer or repressor; low ara, 0.02% arabinose; high ara, 0.2% arabinose. Data are shown as the mean from three biological replicates ± SEM. *, *P* < 0.05 by the nonparametric Mann-Whitney test; ns, *P* > 0.05 by the Mann-Whitney test.

Therefore, we asked whether the effect of ElyC on overall ECA biosynthesis was due to Und-P allocation. We compared the effect of overexpressed *murA*, the first committed step for peptidoglycan biosynthesis (61), with that of the overexpression of *elyC* to determine whether they phenocopy each other. Although overexpression of *murA* does cause a decrease in the abundance of ECA_LPS_, it is much less than that caused by *elyC* (Fig. 5A). Both *murA* and *elyC* decrease levels of linear ECA observed through immunoblotting (Fig. S4B). This result suggests that increasing the competition for substrates may not be solely responsible for the effect of ElyC on ECA biosynthesis. If ElyC did act through balancing Und-P utilization, we further reasoned that overexpression of *wecA*, the first gene in ECA biosynthesis, would suppress the effect of *elyC* overexpression on ECA biosynthesis. To determine whether this was true, we overexpressed both *wecA* and *elyC* in the wild-type strain. We observed that overexpression of *wecA* increased production of ECA_LPS_ when *elyC* was not overexpressed (Fig. 5B, fucose samples). However, there was no increase in ECA_LPS_ levels with *wecA* overexpression when *elyC* was induced with low arabinose or high arabinose levels (Fig. 5B). Therefore, the effect of ElyC on total ECA levels is not through allocation of Und-P.

## DISCUSSION

**FIG 6:**
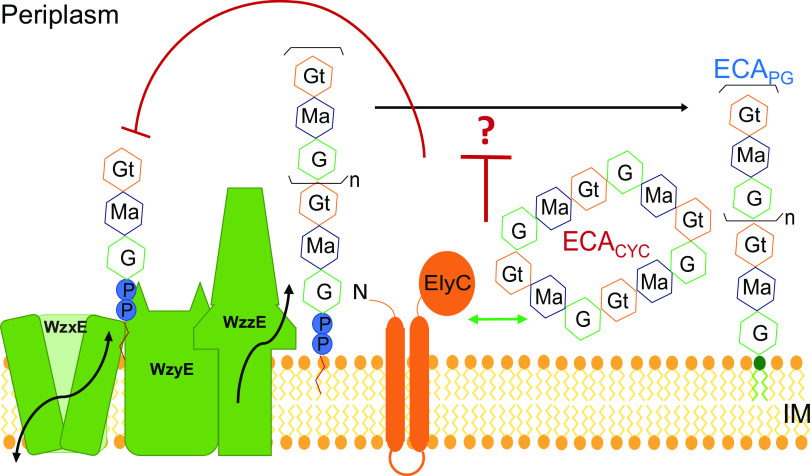
Model for regulation of ECA_PG_ biosynthesis. Our data demonstrate that ElyC and ECA_CYC_ are part of a pathway that regulates the levels of ECA_PG_ and that ElyC can also regulate total ECA levels to a lesser extent. WzxE, WzyE, and WzzE flip completed ECA repeat units on Und-PP across the IM and polymerize the ECA molecule to a regulated chain length. We propose that ElyC and ECA_CYC_ act together to regulate the reaction that removes polymerized ECA from Und-PP and forms phosphoglyceride-linked ECA_PG_. “?” represents the unknown enzymes(s) responsible for this reaction. As ECA_CYC_ is present in the periplasm, its levels can be assessed by ElyC to provide feedback regulation. This assessment may involve a physical interaction.

In this study, we have elucidated a novel pathway regulating the biosynthesis of ECA_PG_ (Fig. 6). We have provided evidence that ElyC specifically regulates the biosynthesis of ECA_PG_ by acting as an inhibitor under normal physiological conditions. Furthermore, we have revealed that full function of ElyC requires the presence of ECA_CYC_ and that ECA_CYC_ itself can regulate the level of ECA_PG_. The mechanism of regulation by both ElyC and ECA_CYC_ appears to be posttranscriptional.

A number of studies have shown that disruption of intermediate steps in ECA biogenesis leads to isoprenoid carrier stress and peptidoglycan synthesis defects due to the accumulation of ECA synthesis intermediates on Und-P (26–30). In fact, a recent study has biochemically confirmed the accumulation of these intermediates (25). Similar defects have been observed with the disruption of O-antigen or colanic acid biosynthesis (60, 83). It is clear that the stress on peptidoglycan synthesis increases the further down the ECA biosynthesis pathway that the disruption occurs (26, 29, 60). Thus, blocking the pathway after the first sugar is added to Und-P causes very little stress, while blocking the pathway after addition of the next sugar causes cell shape defects, stress response activation, and increased permeability, and blocking the pathway after the addition of the third sugar is lethal (26–29, 30).

The possibility that, if only one form of ECA could be made, disruption of a step in ECA biosynthesis past polymerization would also be lethal led us to our TraDIS approach to studying ECA_PG_ biosynthesis. Interestingly, we observed the most robust phenotype from increased ECA_PG_ biosynthesis rather than loss of ECA_PG_ biosynthesis, elucidating another route to sequester Und-P. Under these conditions, the transfer of ECA from Und-PP to make ECA_PG_ may become limiting, causing the buildup of polymerized ECA on Und-PP. Although we are analyzing other hits obtained in the TraDIS experiment, it is likely the phenotype of the Δ*elyC* mutant has a more substantial effect than the loss of ECA_PG_ synthesis itself. In this case, our data would suggest that the Und-PP released by the polymerization of ECA combined with new synthesis of Und-P is sufficient for peptidoglycan synthesis. Recent advances in the biochemical analysis of ECA biosynthesis intermediates (25) will make this an interesting area for future investigation.

With the methods of detecting ECA we have employed, we cannot exclude the possibility that the increase in ECA we observe when deleting *elyC* is due to the accumulation of polymerized ECA on Und-P, suggesting Δ*elyC* disrupts ECA_PG_ biosynthesis. In this model, overexpressing *elyC* would decrease the accumulation of ECA on Und-P, leading to lower levels of ECA observed. However, we do not feel this model fits our data well for the following reasons. (i) The ECA species observed in the immunoblot assay appears to be the major form of ECA present in the cells. To our knowledge, polymerized ECA attached to Und-P has never been detected when Und-P-linked ECA biosynthesis intermediates are investigated (25, 45). In fact, loss of the ability to make a full subunit of ECA was required to initially demonstrate that ECA is synthesized on Und-P (45). (ii) If overexpressing *elyC* increases conversion of Und-P-linked ECA to ECA_PG_, an amount of ECA_PG_ should be produced equal to the amount of Und-P-linked ECA lost, leading to very little change in the amount of detectable linear ECA when *elyC* is overexpressed. (iii) At least some proportion of the increased ECA observed when *elyC* is deleted is surface exposed, and it is unlikely that there is a surface exposure mechanism for Und-P-linked ECA. (iv) Finally, accumulation of Und-P-linked ECA cannot explain the large decrease in linear ECA levels observed when *waaL* is deleted. Deletion of *waaL* prevents the production of ECA_LPS_, removing one possible route for Und-P-linked ECA to be processed to the final forms of ECA; therefore, deletion of *waaL* would not be expected to decrease levels of Und-P-linked ECA.

The synthesis of ECA overall, and of ECA_PG_ in particular, occurs at the IM, a location that is physically distant from the eventual localization of ECA_PG_ at the cell surface (21). Thus, it would be difficult for the biosynthesis of ECA_PG_ to be directly regulated based on the accumulation of ECA_PG_ on the cell surface. ECA_CYC_ provides an ideal solution to this problem, allowing the biosynthesis of ECA to be assessed using a molecule located in the more accessible periplasm (29). As an IM protein with N-terminal transmembrane domains and a comparatively large periplasmic domain (58), ElyC is well situated to interact physically, as well as genetically, both with the ECA biosynthesis machinery and with ECA_CYC_. Studies of ElyC’s physical interactions are ongoing in our lab.

Given their respective localizations, we favor a model in which ElyC provides feedback regulation for the reaction(s) producing ECA_PG_ based on the levels of ECA_CYC_ present in the periplasm (Fig. 6). In this model, we speculate that ElyC undergoes constant transient interactions with ECA_CYC_ that control the activity or binding capability of ElyC. Thus, when ElyC and ECA_CYC_ interact, ElyC would become functional and inhibit the reaction producing ECA_PG_, possibly through direct interaction with the protein(s) responsible for synthesizing ECA_PG_. The inhibition could occur through alteration of activity or of degradation rates of the protein(s) producing ECA_PG_. The amount or time of interaction between ElyC and ECA_CYC_ would, therefore, control how much ECA_PG_ is produced. Levels of ECA_CYC_ could in this way be constantly monitored to maintain appropriate levels of ECA production, while leaving ECA_CYC_ largely free to perform its functional roles in the cell.

In this model, it would be possible for ElyC and ECA_CYC_ to act alone. However, it is also possible that other members of the regulatory pathway exist. These pathway members would not be in our TraDIS data set if their loss did not cause a large increase in ECA_PG_ biosynthesis (e.g., their effects are not inhibitory or they are redundant) or if the genes involved are essential due to their roles in other pathways. If there are other pathway members involved to transmit signals to the cytoplasm, the regulation of ECA_PG_ biosynthesis could also occur by controlling the levels of the protein(s) responsible for ECA_PG_ biosynthesis.

Previous work found deletion of *elyC* causes lysis of cells at room temperature in LB medium with 1% NaCl (LB Miller) (28). This lysis occurred due to a severe defect in peptidoglycan biosynthesis, which was attributed to allocation of Und-P between biosynthesis pathways. The peptidoglycan biosynthesis defect was not observed in cells grown at 37°C (28), the temperature at which our experiments were performed. Nevertheless, our results are consistent with the observation of isoprenoid stress inhibiting peptidoglycan synthesis in Δ*elyC* strains. Specifically, we have determined that this stress is due to the overproduction of ECA_PG_ rather than to the initial allocation of Und-P for peptidoglycan biosynthesis. In fact, our data demonstrate that the effect of ElyC overexpression on total ECA levels is epistatic to the allocation of Und-P between biosynthesis pathways (Fig. 5). Thus, the effect of ElyC on Und-P availability for peptidoglycan synthesis is due to its role in regulating the synthesis of ECA_PG_.

Our data suggest three possible explanations for the more severe phenotype of *elyC* loss at lower temperatures. (i) Disruption or dysregulation of biosynthetic or transport pathways, such as protein secretion, can lead to cold-sensitive phenotypes due to slowing of the pathway at lower temperatures (84). Thus, growth at room temperature might slow ECA synthesis more than peptidoglycan synthesis, leading to increased sequestration of Und-P at lower temperatures. (ii) We have observed the chain length (number of repeat units per molecule) of ECA is less at 30°C than at 37°C (36). Thus, the same amount of ECA repeat units will be made into more final ECA molecules, increasing the amount of Und-P utilized for ECA synthesis at lower temperatures. (iii) Finally, ElyC may have an additional function at room temperature that also diverts Und-P from peptidoglycan synthesis that is not apparent at 37°C. Interestingly, Kouidmi et al. found an increase in periplasmic protein aggregation when Δ*elyC* cells were grown at room temperature that could be suppressed by overproduction of two periplasmic chaperones, DsbG and Spy, leading to restoration of peptidoglycan biosynthesis (69). These data may suggest an additional function for ElyC during growth at low temperatures.

The three forms of ECA are synthesized from a common precursor—polymerized ECA on Und-PP. Clearly, mechanisms are necessary to ensure the proper balance is maintained between the forms of ECA, both to support their proper functions and to avoid stress caused by dysregulation of biosynthetic pathways. WaaL, the O-antigen ligase, attaches both ECA and O-antigen to LPS (43, 85). In smooth strains that produce O-antigen, very little ECA_LPS_ is produced (43, 85, 86), suggesting that the availability of WaaL is a rate-limiting step in the production of ECA_LPS_. Our results confirm that increasing the levels of WaaL causes more ECA_LPS_ to be produced. Our data further suggest the regulation of production of ECA_PG_ and ECA_CYC_ is dependent on feedback regulation by ElyC based on ECA_CYC_ levels. The lesser effect of this regulatory pathway on ECA_LPS_ may suggest that ECA_LPS_ production is largely a by-product of O-antigen synthesis. In many *Enterobacterales*, O-antigen shares an initial GlcNAc residue with ECA (43, 87, 88), which may lead WaaL to be somewhat promiscuous. Nevertheless, surface exposure of ECA_LPS_ leads to production of ECA antibodies, the consequences of which have not been fully explored (21).

Since the discovery of ECA_CYC_, there has been longstanding debate about whether ECA_CYC_ has a role in biosynthesis of the other forms of ECA or plays its own functional role within the cell. Previously, we demonstrated ECA_CYC_ plays a role in maintaining the OM permeability barrier (36). In our current work, we show that ECA_CYC_ also plays a role in regulating the synthesis of ECA_PG_. Thus, our work clearly indicates that ECA_CYC_ plays a dual role in the cell—both necessary for the proper function of the OM permeability barrier and involved in the regulation of ECA synthesis. This can be compared to classic biosynthetic pathways, such as those for amino acid synthesis, where the product of the pathway, useful in and of itself, also acts to allosterically regulate its own production, maintaining a constant pool of the biosynthetic product (89). Overall, the discovery of the ElyC-ECA_CYC_ pathway controlling ECA_PG_ biosynthesis will provide a foothold in characterization of the mechanism of ECA_PG_ biosynthesis, in understanding the regulation of ECA synthesis under changing environmental conditions, and in investigating both the functional and regulatory role of ECA_CYC_.

## MATERIALS AND METHODS

### Bacterial strains and plasmids.

Bacterial strains, plasmids, and primers used in this study are listed in Table S4 in the supplemental material. Strains were grown at 37°C in LB Lennox medium with the necessary antibiotics: kanamycin (25 mg/liter), chloramphenicol (20 mg/liter), and tetracycline (20 mg/liter), unless otherwise noted. IPTG at the indicated concentrations (0 to 100 μM) was used for overexpression from the pCA24N plasmid. l-Arabinose and α-d-fucose at the indicated concentrations were used to induce or repress the P_BAD_ promoter, respectively. The deletion alleles were utilized from the Keio collection (65), unless otherwise indicated. New deletion alleles were constructed using λ-Red recombineering, as has been described previously (90). Mutants were made by P1*vir* transduction (91). Markerless deletion strains were generated by flipping out the kanamycin cassette with Flp recombinase as described previously (90).

10.1128/mBio.02846-21.10 TABLE S4 Bacterial strains, plasmids, and primers. Download Table S4, PDF file, 0.2 MB. Copyright © 2021 Rai et al. 2021 Rai et al. https://creativecommons.org/licenses/by/4.0/ This content is distributed under the terms of the Creative Commons Attribution 4.0 International license.

Plasmids from the ASKA library were used for overexpression experiments (79). *elyC* with its native ribosome binding site (RBS) was cloned into pBAD33 (92) through HiFi Assembly (New England Biolabs [NEB]) per the manufacturer’s protocol using the pBAD33 and *elyC* (o/l pBAD33) primers (Table S4). Subsequently, the chloramphenicol resistance cassette was replaced with the kanamycin resistance cassette from pZS21 (93) using HiFi assembly and the pBAD33-*elyC* and kanR primers (Table S4). The promoter region of the *wec* operon was cloned from −500 to +20 in relation to the start codon of *wecA* upstream of the *luxCDABE* operon in the pJW15 vector (80, 81) using HiFi assembly and the Pwec and pNLP10/JW15 primers (Table S4).

### TraDIS sample preparation and analysis.

Transposon mutant libraries were constructed from Δ*wzzE* Δ*waaL* and Δ*wecA* Δ*wzzE* Δ*waaL* strains by electroporation of the EZ-Tn*5* <KAN-2>Tnp transposome (Lucigen) as previously described (94). The library in wild-type MG1655 was previously described (94). About 306,000 and 186,000 individual colonies were pooled for the initial transposon library of the Δ*wzzE* Δ*waaL* and Δ*wecA* Δ*wzzE* Δ*waaL* strains, respectively. Liquid LB cultures were grown from the pooled libraries of mutants for 10 generations. DNA was extracted from the pooled libraries before and after growth in liquid medium using the DNeasy blood and tissue kit (Qiagen) according to the manufacturer’s instructions. Next, Illumina DNA fragment libraries were prepared using the TraDIS approach and sequenced on an Illumina HiSeq 2500 sequencer, as has been described previously (64, 94). Data were analyzed and mapped to the E. coli K-12 genome NC_000913.3 as has been described previously (94).

### Analysis of ECA levels. (i) Immunoblot analysis for linear ECA levels.

ECA levels were assayed from overnight cultures, as previously described with slight modifications (36). Specifically, membranes were probed with a rabbit polyclonal anti-ECA antibody at a 1:30,000 dilution (a kind gift from Renato Morona at the University of Adelaide). Goat anti-rabbit secondary antibody conjugated to horseradish peroxidase (Prometheus) was used at a 1:100,000 dilution and detected using Prosignal Pico ECL (Prometheus) using Prosignal enhanced chemiluminescence (ECL) blotting film (Prometheus).

### (ii) Dot blot for ECA surface exposure.

Surface-exposed ECA was detected using a dot blot assay as has been described previously with minor modifications (72). Specifically, the following antibodies were used for detection: anti-ECA (1:5,000), anti-BamD (1:5,000), and anti-RcsF (1:5,000) (95, 96).

### (iii) WGA staining for ECA_LPS_ quantification.

Standard conditions for WGA staining of ECA_LPS_ were as follows. Two hundred fifty microliters of overnight culture was centrifuged for 3 min at 3,400 × *g* in round-bottom 96-well plates. After removing the supernatant, pelleted cells were washed with 200 μl of 1× phosphate-buffered saline (PBS). After washing, cells were resuspended in 200 μl of 1× PBS with a 1:100 volume of WGA-AF488 (Thermo Fisher Scientific) prepared per the manufacturer’s instructions. Samples were incubated in the dark at room temperature for 10 min. Then, cells were washed twice with 200 μl 1× PBS and resuspended in 110 μl 1× PBS. Next, 100 μl of each sample was aliquoted to a black-wall, clear-bottom 96-well plate where the optical density at 600 nm (OD_600_) and fluorescence at excitement (Ex.) 485 nm and emission (Em.) 519 nm was recorded using a BioTek Synergy H1 plate reader autogained based on sample fluorescence. Fluorescence relative to OD_600_ was calculated.

### (iv) Quantification of ECA_CYC_.

For ECA_CYC_ quantification, ECA_CYC_ was purified and subjected to matrix-assisted laser desorption ionization–time of flight (MALDI-TOF) mass spectrometry, and the relative abundance of ECA_CYC_ between samples was calculated as has been described previously (29, 36).

### P*_wec_* reporter assay.

Overnight cultures of the indicated strains containing the pJW15-P*_wec_* plasmid were subcultured (1:100) into 100 μl of fresh LB broth in a black-wall, clear-bottom 96-well plate. The plate was sealed with a Breathe-Easy sealing membrane (Sigma), and the luminescence and OD_600_ of each strain were measured every 3 min for 6 h using a BioTek Synergy H1 plate reader as previously described (80, 81). Each biological replicate was performed in technical quadruplicate.

### Data availability.

The sequencing data are available in the Sequence Read Archive database (SRA) (https://www.ncbi.nlm.nih.gov/sra, BioProject ID PRJNA763934).
