# Supplementary figures and images for: ElyC and Cyclic Enterobacterial Common Antigen Regulate Synthesis of Phosphoglyceride-Linked Enterobacterial Common Antigen
Source: mBio. 2021 Nov 23;12(6):e02846-21. doi: 10.1128/mBio.02846-21 (PMC8609368; doi:10.1128/mBio.02846-21)

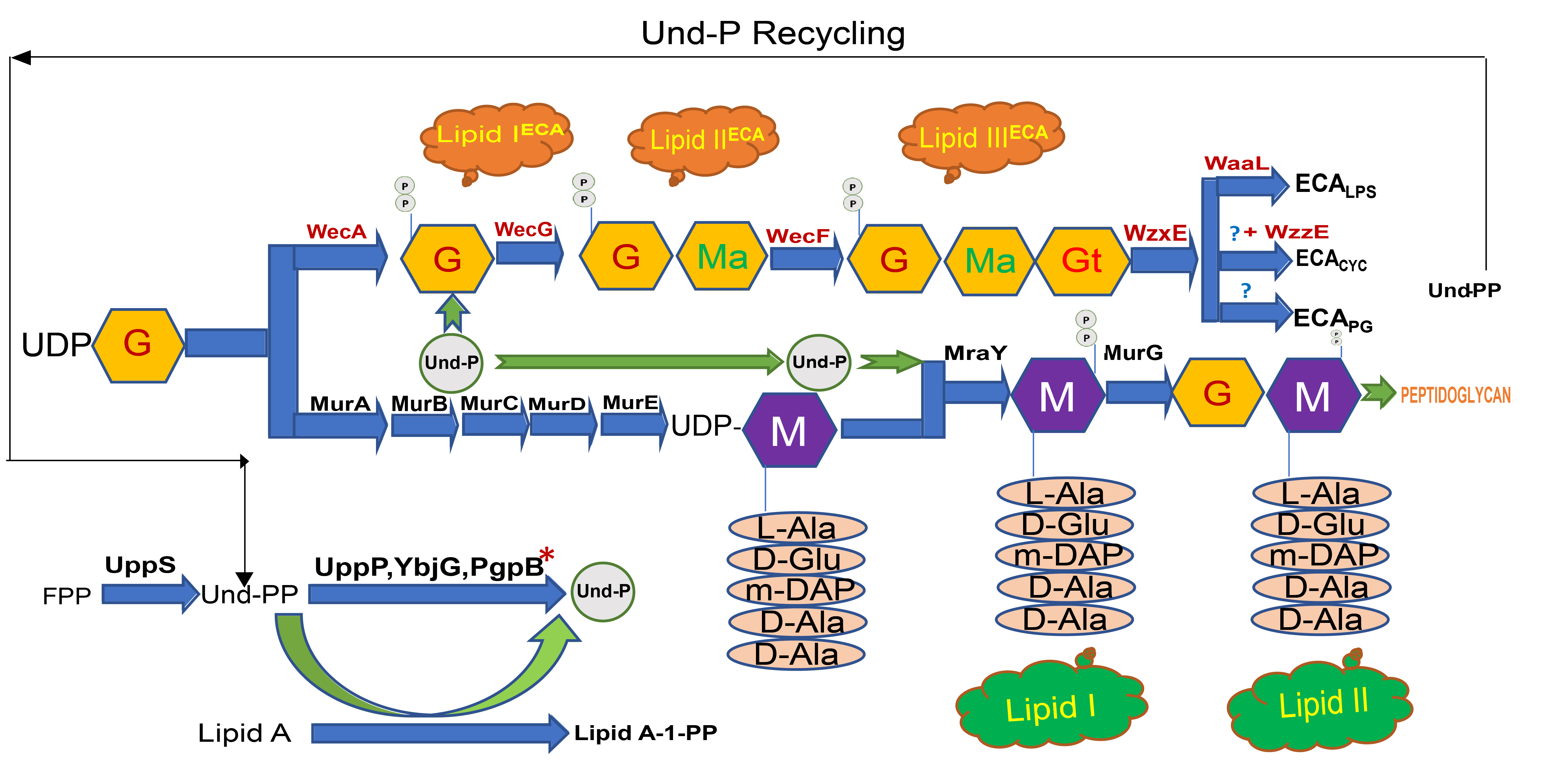

Supplement: FIG S1 [file mbio.02846-21-sf001.jpg]

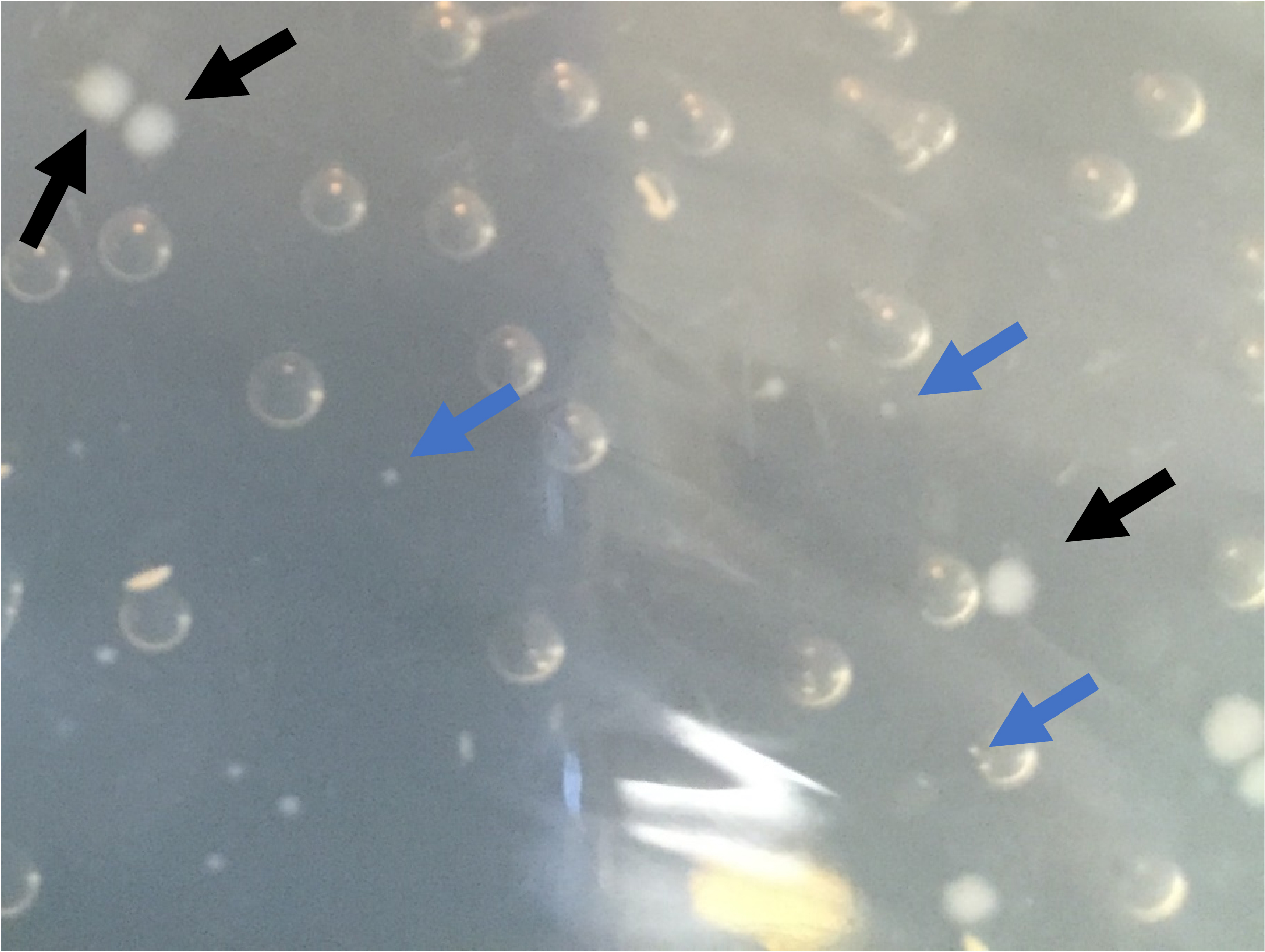

Supplement: FIG S2 [file mbio.02846-21-sf002.jpg]

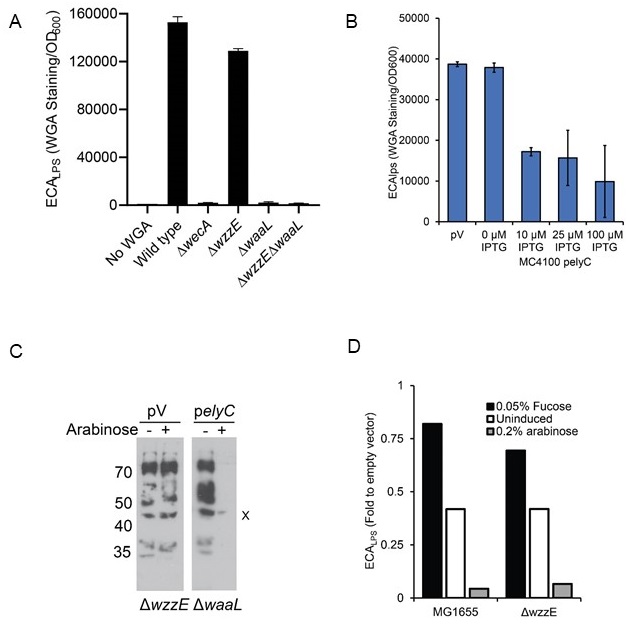

Supplement: FIG S3 [file mbio.02846-21-sf003.jpg]

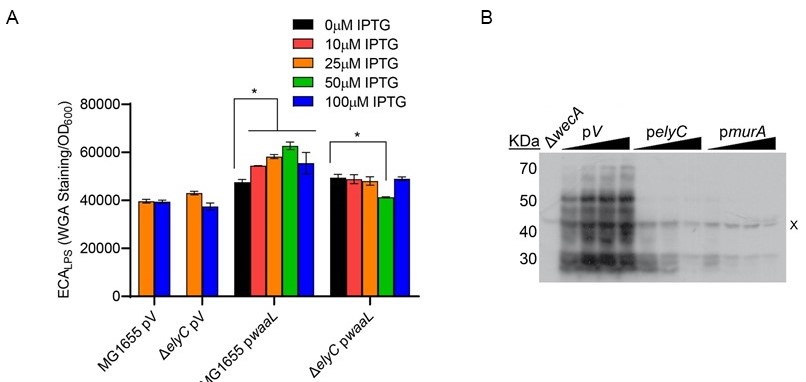

Supplement: FIG S4 [file mbio.02846-21-sf004.jpg]

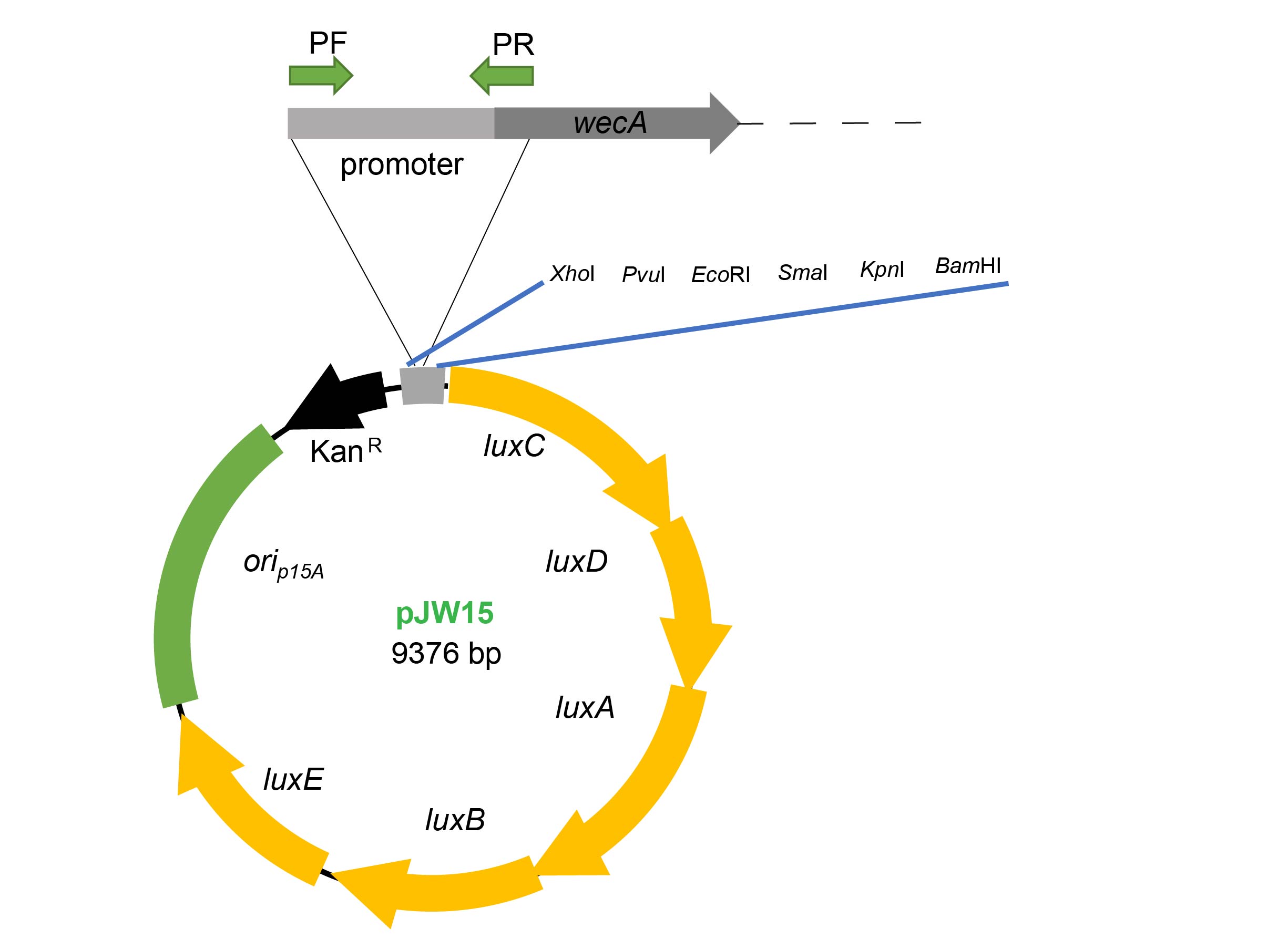

Supplement: FIG S5 [file mbio.02846-21-sf005.jpg]

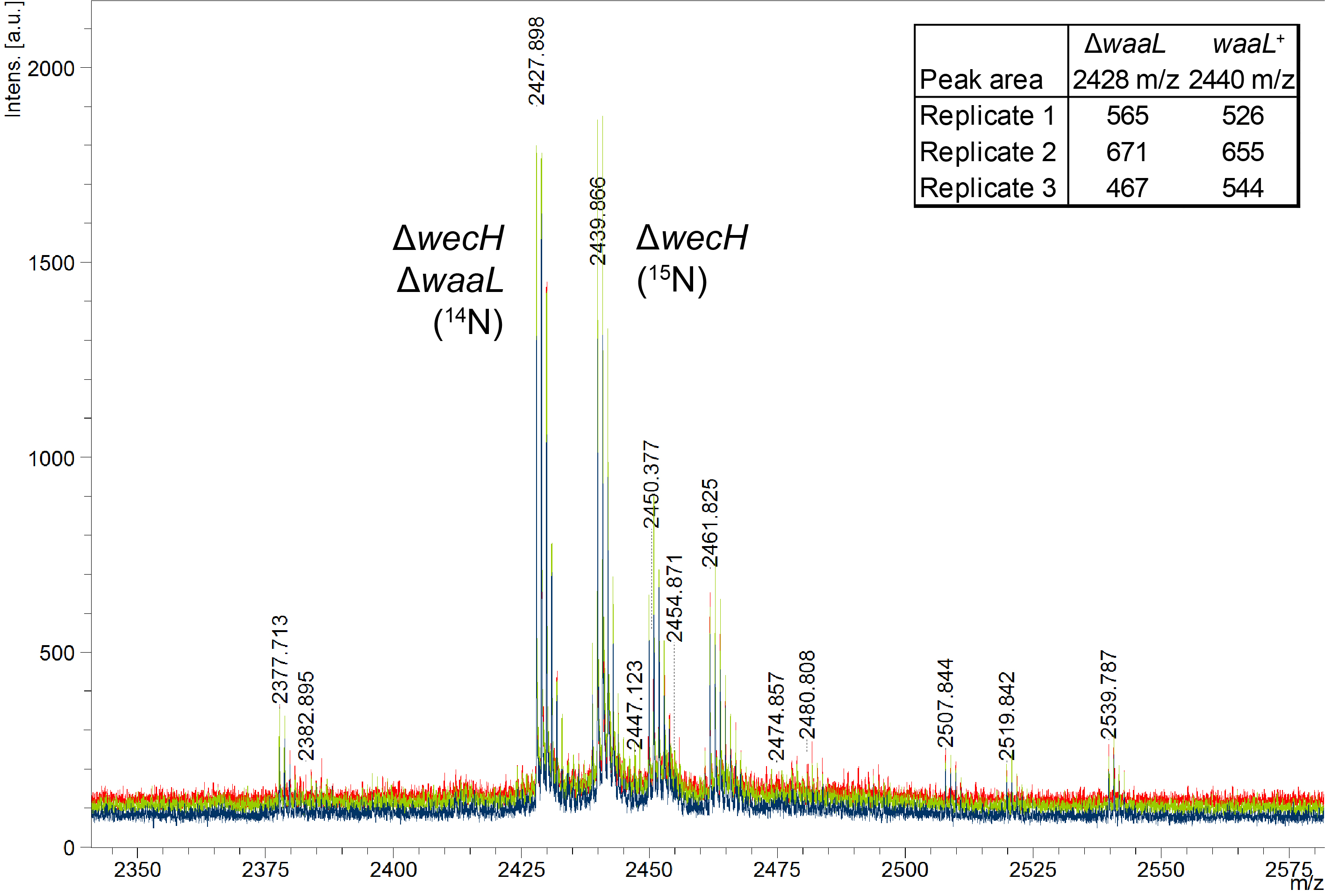

Supplement: FIG S6 [file mbio.02846-21-sf006.jpg]
