# Supplementary material for: ElyC and Cyclic Enterobacterial Common Antigen Regulate Synthesis of Phosphoglyceride-Linked Enterobacterial Common Antigen
Source: mBio. 2021 Nov 23;12(6):e02846-21. doi: 10.1128/mBio.02846-21 (PMC8609368; doi:10.1128/mBio.02846-21)
Supplement: TABLE S1 [file mbio.02846-21-st001.pdf]

Table S1: Descriptive statistics for TraDIS

	Reads per gene for given samples[a]					
	**AM395-T=0 h**	**MG1655-T=0 h**	**AM652-T=0 h**	**AM395-T=5 h**	**MG1655-T=5 h**	**AM652-T=5 h**
**Mean**	7975	7799	8944	7627	9631	7105
**Median**	5021	4799	5753	3936	4567	3669

	Log 2-fold values between indicated samples[a]			
	**AM395/MG1655 T=0 h**	**AM395/AM652 T=0 h**	**AM395/MG1655 T=5 h**	**AM395/AM652 T=5 h**
**Mean**	0.20	-0.24	-0.02	0.03
**Median**	0.09	-0.18	-0.19	0.06
**Std Dev**	1.64	1.27	1.92	1.34
**Maximum**	11.7	9.1	10.9	9.8
**Minimum**	-11.2	-10.7	-11.3	-10.4

[a] The indicated strains are wild type (MG1655), a strain with only ECA_{PG} (AM395), and an isogenic strain with no ECA (AM652). T=0 h corresponds to libraries grown on plates, while T=5 h corresponds to libraries after 10 generations of growth in liquid media.
