# Supplementary material for: ElyC and Cyclic Enterobacterial Common Antigen Regulate Synthesis of Phosphoglyceride-Linked Enterobacterial Common Antigen
Source: mBio. 2021 Nov 23;12(6):e02846-21. doi: 10.1128/mBio.02846-21 (PMC8609368; doi:10.1128/mBio.02846-21)
Supplement: TABLE S2 [file mbio.02846-21-st002.pdf]

Table S2: Possible genes involved in ECA_{PG} biogenesis

	Reads per Kb[b]			Log 2-fold values[c]	
**Gene[a]**	**AM395**	**MG1655**	**AM652**	**AM395/MG1655**	**AM395/AM652**
**Growth on Plates**					
elyC	137	1938	4455	-3.8	-5.0
ynbB	82	2621	776	-5.0	-3.2
ymiB	5	990	4210	-7.7	-9.8
lapA	430	7790	1524	-4.2	-1.8
yoaI	1819	9429	6162	-2.4	-1.8
**10 generations in liquid media**					
elyC	101	944	2919	-3.2	-4.8
ynbB	77	1594	367	-4.4	-2.3
ymiB	5	1210	1933	-8.0	-8.7
lapA	6	2544	741	-8.6	-6.8
yoaI	476	6048	2810	-3.7	-2.6

[a] Genes fitting the following criteria for possible roles in ECA_{PG} biosynthesis. (1) Not known to be essential in wild type *E. coli* K-12, (2) Gene product is an inner membrane or periplasmic protein, (3) Less than 200 un-normalized reads in ECA_{PG}, (4) More than one standard deviation fold decrease in ECA_{PG} strain compared to other strains in both growth conditions

[b] Read counts assigned to the indicated genes normalized to gene length in libraries in wild type (MG1655), a strain with only ECA_{PG} (AM395), and an isogenic strain with no ECA (AM652).

[c] Log2 fold values comparing the read counts per gene between the indicated libraries.
