# Supplementary material for: ElyC and Cyclic Enterobacterial Common Antigen Regulate Synthesis of Phosphoglyceride-Linked Enterobacterial Common Antigen
Source: mBio. 2021 Nov 23;12(6):e02846-21. doi: 10.1128/mBio.02846-21 (PMC8609368; doi:10.1128/mBio.02846-21)
Supplement: TABLE S3 [file mbio.02846-21-st003.pdf]

Table S3: Linkage disruption with deletion of *waaL*

Donor	Recipient	Recipient forms of ECA	N[a]	P1 Linkage frequency[b]
tdh::Tn*10* Δ*waaL*::kan (AM735)	MG1655	ECA_{CYC}, ECA_{LPS}, ECA_{PG}	154	79%
	Δ*elyC* (AM743)	ECA_{CYC}, ECA_{LPS}, ECA_{PG}	100	80%
	Δ*elyC*::cm (AM1045)	ECA_{CYC}, ECA_{LPS}, ECA_{PG}	208	75%
	Δ*wzzE* (AM365)	ECA_{LPS}, ECA_{PG}	46	70%
	Δ*wzzE*—rebuilt (AM1121)	ECA_{LPS}, ECA_{PG}	100	77%
	Δ*wzzE* Δ*elyC*::cm (AM1047)	ECA_{LPS}, ECA_{PG}	59	75%
	Δ*wzzE*—rebuilt Δ*elyC*::cm (AM1125)	ECA_{LPS}, ECA_{PG}	199	60%
	Δ*elyC* Δ*wzzE* (AM1123)	ECA_{LPS}, ECA_{PG}	200	49%

[a] Number of transductants analyzed

[b] Indicated markers were transduced into the indicated strains using P1*vir*. Linkage frequency indicates percentage of strains transduced with *thd*::Tn*10* also containing Δ*waaL*::kan.
