# Supplementary material for: ElyC and Cyclic Enterobacterial Common Antigen Regulate Synthesis of Phosphoglyceride-Linked Enterobacterial Common Antigen
Source: mBio. 2021 Nov 23;12(6):e02846-21. doi: 10.1128/mBio.02846-21 (PMC8609368; doi:10.1128/mBio.02846-21)
Supplement: TABLE S4 [file mbio.02846-21-st004.pdf]

**Table S4: Bacterial strains, plasmid, and primers**

| STRAIN | GENOTYPE | REFERENCE | Description |
|---|---|---|---|
| MG1655 | K-12 $F^-$ $\lambda^-$ *rph-1* | (1) | |
| AM395 | MG1655 Δ*wzzE* Δ*waaL* | (2) | |
| AM652 | MG1655 Δ*waaL* Δ*wecA-wzzE* | This study | |
| AM334 | MG1655 Δ*wecA* | (2) | |
| AM365 | MG1655 Δ*wzzE* | (2) | |
| AM366 | MG1655 Δ*waaL* | (2) | |
| AM743 | MG1655 Δ*elyC* | This study | |
| AM744 | MG1655 Δ*wzzE* Δ*elyC* | This study | |
| AM745 | MG1655 Δ*waaL* Δ*elyC* | This study | |
| AM735 | MG1655 Δ*waaL*::kan *tdh*::Tn10 | This study | Tn10 allele from Grabowicz, *et al.* 2014 (3) |
| AM766 | MG1655 Δ*wzzE*::kan *metE*-3074::Tn10 | This study | Tn10 allele from Singer, *et al.* 1989 (4) |
| AM769 | MG1655 Δ*elyC*::kan *zbj*-7230::Tn10 | This study | Tn10 allele from Singer, *et al.* 1989 (4) |
| AM1045 | MG1655 Δ*elyC*::cm | This study | Newly recombineered *elyC* deletion allele |
| AM1047 | MG1655 Δ*wzzE* Δ*elyC*::cm | This study | |
| AM1121 | MG1655 Δ*wzzE* | This study | Rebuilt from MG1655 |
| AM1125 | MG1655 Δ*wzzE* Δ*elyC*::cm | This study | Built from AM1121 |
| AM1123 | MG1655 Δ*elyC* Δ*wzzE* | This study | |
| AM397 | MG1655 Δ*wecH*::kan | (2) | |
| AM518 | MG1655 Δ*waaL* Δ*wecH*::kan | This study | |
| AM1138 | MG1655 pCA24N | This study | Contains ASKA collection empty plasmid (5), $cm^R$ |
| AM1139 | MG1655 pCA24N-*elyC* | This study | Plasmid from ASKA collection (5) |
| AM1140 | MG1655 Δ*wzzE* pCA24N | This study | |
| AM1141 | MG1655 Δ*wzzE* pCA24N-*elyC* | This study | |
| AM1142 | MG1655 Δ*waaL* pCA24N | This study | |
| AM1143 | MG1655 Δ*waaL* pCA24N-*elyC* | This study | |
| AM1219 | MG1655 pCA24N-*murA* | This study | Plasmid from ASKA collection (5) |
| AM1162 | MG1655 pBAD33 | This study | Plasmid from Guzman, *et al.* 1995 (6), $cm^R$ |
| AM1163 | MG1655 pBAD33-*elyC* | This study | *elyC* cloned into pBAD33 with native ribosome binding site |
| AM1166 | MG1655 Δ*wzzE* pBAD33 | This study | |
| AM1167 | MG1655 Δ*wzzE* pBAD33-*elyC* | This study | |
| AM1158 | MG1655 Δ*wzzE* Δ*waaL* pBAD33 | This study | |
| AM1159 | MG1655 Δ*wzzE* Δ*waaL* pBAD33-*elyC* | This study | |
| AM1196 | MG1655 pCA24N-*waaL* | This study | Plasmid from ASKA collection (5) |

| | | | |
|---|---|---|---|
| AM1197 | MG1655 Δ*elyC* pCA24N | This study | |
| AM1198 | MG1655 Δ*elyC* pCA24N-*waaL* | This study | |
| AM1199 | MG1655 pBAD33(K)-*elyC* pCA24N | This study | pBAD33(K)-*elyC* is pBAD33-*elyC* with $cm^R$ cassette switched to $kan^R$ cassette |
| AM1223 | MG1655 pBAD33(K)-*elyC* pCA24N-*wecA* | This study | *wecA* plasmid from ASKA collection (5) |
| AM944 | MG1655 pJW15 ($kan^R$) | This study | Plasmid from MacRitchie, *et al.* 2008 (7) |
| AM1225 | MG1655 pJW15- $P_{wec}$ | This study | Promoter of *wec* operon cloned into pJW15 (see **Fig. S5**) |
| AM1226 | MG1655 Δ*wecA* pJW15-$P_{wec}$ | This study | |
| AM1227 | MG1655 Δ*wzzE* pJW15-$P_{wec}$ | This study | |
| AM1228 | MG1655 Δ*waaL* pJW15-$P_{wec}$ | This study | |
| AM1229 | MG1655 Δ*elyC* pJW15-$P_{wec}$ | This study | |
| AM1237 | MG1655 pCA24N pJW15-$P_{wec}$ | This study | |
| AM1238 | MG1655 pCA24N-*elyC* pJW15-$P_{wec}$ | This study | |
| MC4100 | F-*araD139* (*argF-lac*)*U169 rpsL150 relA1 flb5301 deoC1 ptsF25 thi* | (8) | |
| JCM158 | MC4100 $Ara^{R/-}$ | (9) | |
| AM1271 | JCM158 pCA24N | This study | |
| AM1272 | JCM158 pCA24N-*elyC* | This study | |

| **Primer** | **Primer 5′ (overlap/spacer/ANNEAL) 3′** |
|---|---|
| pBAD33 F | CCGGGGATCCTCTAGAGTC |
| pBAD33 R | GTACCGAGCTCGAATTCG |
| elyC (o/l pBAD33) F | agcgaattcgagctcggtacGCTATATGGTTAAAAAGGAACCAGAAAG |
| elyC (o/l pBAD33) R | cgactctagaggatccccggTCACTCTTGCCGTGGCTC |
| pBAD33-elyC_fwd | CGAAGTGATCTTCCGTCAC |
| pBAD33-elyC_rev | CAAATTCGACCCGGTCGTC |
| kanR_fwd | cgacgaccgggtcgaatttgCTCGAACCCCAGAGTCCC |
| kanR_rev | tgtgacggaagatcacttcgGGTGAAGACGAAAGGGCC |
| Pwec F | GAGTTTAAAGGTACAGGC |
| Pwec(S) R | CTCACTGTCAGTAAATTCAC |
| pNLP10/JW15Fo/lPwec(S) | gtgaatttactgacagtgagGCAGCCCGGGTACCGGAT |
| pNLP10/JW15 R o/l Pwec | ttgcctgtacctttaaactcAGAATTCCGATCGCTCGAGGTG |

## REFERENCES


1. Guyer MS, Reed RR, Steitz JA, Low KB. 1981. Identification of a sex-factor-affinity site in E. coli as gamma delta. Cold Spring Harb Symp Quant Biol 45 Pt 1:135-40.
2. Mitchell AM, Srikumar T, Silhavy TJ. 2018. Cyclic Enterobacterial Common Antigen Maintains the Outer Membrane Permeability Barrier of *Escherichia coli* in a Manner Controlled by YhdP. mBio 9:e01321-18.

3. Grabowicz M, Andres D, Lebar MD, Malojcic G, Kahne D, Silhavy TJ. 2014. A mutant *Escherichia coli* that attaches peptidoglycan to lipopolysaccharide and displays cell wall on its surface. elife 4:e05334.
4. Singer M, Baker TA, Schnitzler G, Deischel SM, Goel M, Dove W, Jaacks KJ, Grossman AD, Erickson JW, Gross CA. 1989. A collection of strains containing genetically linked alternating antibiotic resistance elements for genetic mapping of Escherichia coli. Microbiol Rev 53:1-24.
5. Kitagawa M, Ara T, Arifuzzaman M, Ioka-Nakamichi T, Inamoto E, Toyonaga H, Mori H. 2005. Complete set of ORF clones of *Escherichia coli* ASKA library (a complete set of E. coli K-12 ORF archive): unique resources for biological research. DNA Res 12:291-9.
6. Guzman LM, Belin D, Carson MJ, Beckwith J. 1995. Tight regulation, modulation, and high-level expression by vectors containing the arabinose PBAD promoter. J Bacteriol 177:4121-30.
7. MacRitchie DM, Ward JD, Nevesinjac AZ, Raivio TL. 2008. Activation of the Cpx Envelope Stress Response Down-Regulates Expression of Several Locus of Enterocyte Effacement-Encoded Genes in Enteropathogenic *Escherichia coli*. Infection and Immunity 76:1465-1475.
8. Silhavy TJ, Berman ML, Enquist LW. 1984. Experiments with gene fusions. Cold Spring Harbor Laboratory.
9. Malinverni JC, Werner J, Kim S, Sklar JG, Kahne D, Misra R, Silhavy TJ. 2006. YfiO stabilizes the YaeT complex and is essential for outer membrane protein assembly in *Escherichia coli*. Mol Microbiol 61:151-64.
